# Transcriptomic analysis reveals myometrial topologically associated domains linked to the onset of human term labour

**DOI:** 10.1093/molehr/gaac003

**Published:** 2022-02-12

**Authors:** Sonika Tyagi, Eng-Cheng Chan, Daniel Barker, Patrick McElduff, Kelly A Taylor, Carlos Riveros, Esha Singh, Roger Smith

**Affiliations:** 1 Department of Infectious Diseases, Central Clinical School, Monash University and the Alfred Hospital, Melbourne, VIC, Australia; 2 Mothers and Babies Research Centre, HMRI University of Newcastle, NSW, Australia; 3 University of Newcastle, Newcastle, NSW, Australia; 4 Department of Biotechnology and Biochemical Engineering, Indian Institute of Technology, New Delhi, India

**Keywords:** pregnancy, estrogen, parturition, topologically associated domains, epigenome, bioinformatics

## Abstract

Changes in cell phenotype are thought to occur through the expression of groups of co-regulated genes within topologically associated domains (TADs). In this paper, we allocate genes expressed within the myometrium of the human uterus during the onset of term labour into TADs. Transformation of the myometrial cells of the uterus into a contractile phenotype during term human labour is the result of a complex interaction of different epigenomic and genomic layers. Recent work suggests that the transcription factor (TF) RELA lies at the top of this regulatory network. Using deep RNA sequencing (RNAseq) analysis of myometrial samples (n = 16) obtained at term from women undergoing caesarean section prior to or after the onset of labour, we have identified evidence for how other gene expression regulatory elements interact with TFs in the labour phenotype transition. Gene set enrichment analysis of our RNAseq data identified three modules of enriched genes (M1, M2 and M3), which in gene ontology studies are linked to matrix degradation, smooth muscle and immune gene signatures, respectively. These genes were predominantly located within chromosomal TADs suggesting co-regulation of expression. Our transcriptomic analysis also identified significant differences in the expression of long non-coding RNAs (lncRNA), microRNAs (miRNA) and TFs that were predicted to target genes within the TADs. Additionally, network analysis revealed 15 new lncRNA (MCM3AP-AS1, TUG1, MIR29B2CHG, HCG18, LINC00963, KCNQ1OT1, NEAT1, HELLPAR, SNHG16, NUTM2B-AS1, MALAT1, PSMA3-AS1, GABPB1-AS1, NORAD and NKILA) and 4 miRNA (*mir-145*, *mir-223*, *mir-let-7a* and *mir-132*) as top gene hubs with three TFs (NFKB1, RELA and ESR1) as master regulators. Together, these factors are likely to be involved in co-regulatory networks driving a myometrial transformation to generate an estrogen-sensitive phenotype. We conclude that lncRNA and miRNA targeting the estrogen receptor 1 and nuclear factor kappa B pathways play a key role in the initiation of human labour. For the first time, we perform an integrative analysis to present a multi-level genomic signature made of mRNA, non-coding RNA and TFs in the myometrium for spontaneous term labour.

## Introduction

Higher-order chromatin structure and organization is emerging as a key factor in determining how *cis*-regulatory elements work to generate a phenotype or disease risk (Lupiáñez *et al.*, 2015; Franke *et al.*, 2016; Merienne *et al.*, 2019; Liang *et al.*, 2020). Along the linear DNA axis, there are domains of DNA that tend to interact with each other more frequently than with areas outside the interacting domains: these are termed topologically associated domains (TADs; Hou *et al.*, 2012; Nora *et al.*, 2012; Sexton *et al.*, 2012). Evolutionary conservation of TADs between species and cell types suggests their functional relevance (Dixon *et al.*, 2015). TADs are important features of genome organization not only to facilitate chromatin packing but also to restrict enhancer–promoter interactions to the TAD, and breaking of TAD boundaries has pathological consequences (Lupiáñez *et al.*, 2015; Merienne *et al.*, 2019). Recent work on the transformation of cell phenotypes has emphasized the role of co-regulation of groups of genes within TADs to effect major changes in the structure and behaviour of cells (Phillips-Cremins *et al.*, 2013). Higher resolution analysis of TADs has shown that TADs have hierarchical structures forming sub-TAD structures, also known as chromatin loops (Rao *et al.*, 2014). Further, *CTCF* or CCCTC-binding factor is an evolutionary conserved and multifunctional protein that appears to have a role in insulating the TAD boundaries to constrain enhancer–promoter interactions within the domains (Narendra *et al.*, 2015).

Successful human parturition requires the uterus to undergo a programme of transformation from a quiescent expandable receptacle into an active organ able to produce coordinated forceful phasic contractions to push the foetus through a softened cervix to birth (Smith *et al.*, 2012, 2013, 2019, 2020; Kota *et al.*, 2013; Smith, 2015). An understanding of the programme at the molecular level is central to developing precision obstetric interventions for at-risk pregnancies, including to stop premature delivery, successfully induce normal vaginal delivery or to promote uterine contraction to arrest post-partum haemorrhage (Smith *et al.*, 2019). With practical and ethical constraints on experimental approaches and an absence of applicable animal models for human parturition, efforts to understand the programme have focused on mathematical modelling of key variables from single samples of human myometrium obtained at caesarean section.

**Figure 1:**
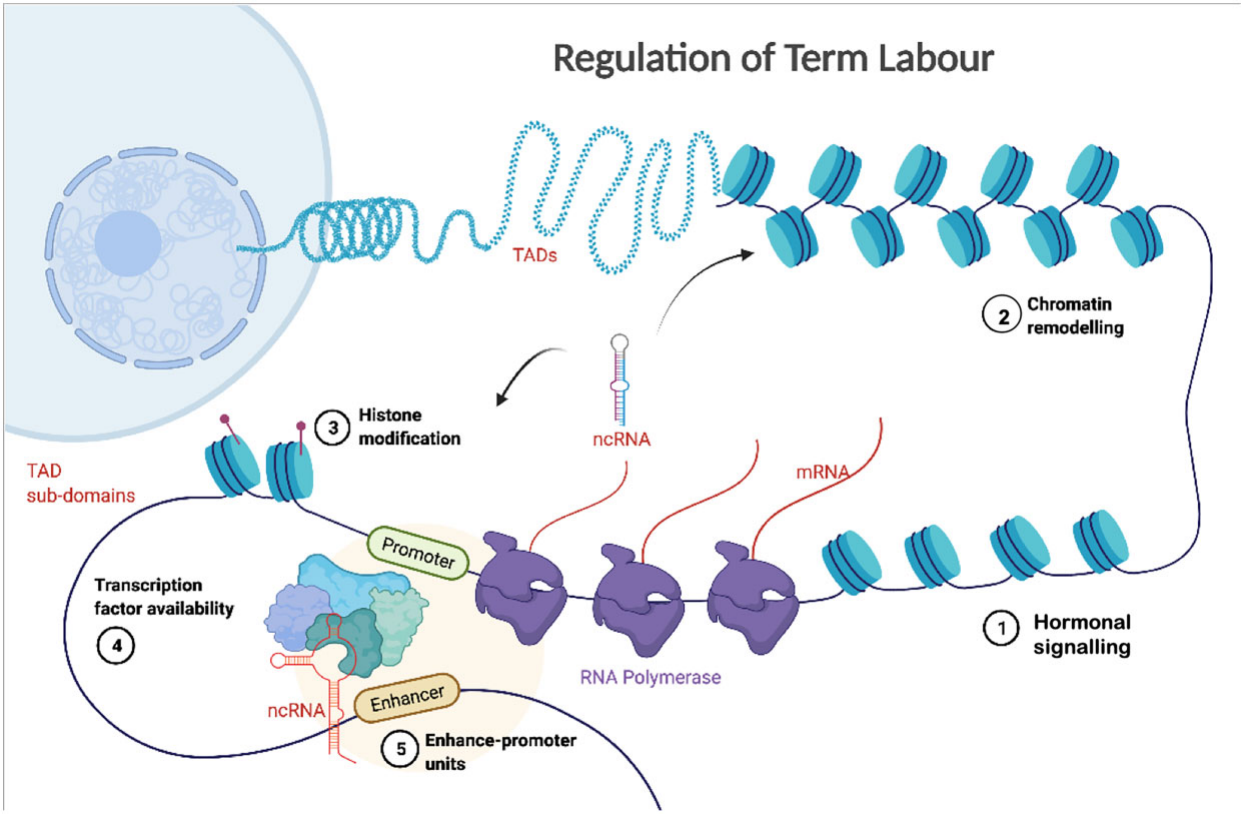
A holistic view of the hierarchical changes in the genome and epigenome to regulate onset of labour at term.

We and others have interrogated genomic and transcriptomic data from term human myometrium to gain insight into the process of labour in parturition. Previously, several studies have employed gene expression profiling using microarray (Esplin *et al.*, 2005; Bukowski *et al.*, 2006; Mittal *et al.*, 2010; Romero *et al.*, 2014; Sharp *et al.*, 2016; Lui *et al.*, 2018) or high-throughput RNA sequencing (RNAseq) to identify genes that are differentially expressed during parturition (Ackerman *et al.*, 2018; Stanfield *et al.*, 2019). These studies have generated a long list of genes that are altered during the process of labour (Stanfield *et al.*, 2019). Although myometrial contractility has been associated with a characteristic gene expression pattern, finding the key drivers from the long differentially expressed list is challenging. We postulated that an interaction of epigenomic factors, including non-coding RNA (ncRNA), and specific gene regulation control determines the transcriptional phenotype. We have examined the hierarchical architecture of the genomic components to identify likely apical drivers of large-scale transcriptional changes within specific TADs (Fig. 1).

## Materials and methods

### Ethics approval

The samples were collected under our University of Newcastle John Hunter Hospital, Newcastle, Australia and the Singapore KK Women’s and Children’s Hospital ethics committees (Ethics approval H3820602). Pregnant women gave written informed consent to donate a biopsy of their myometrium at caesarean section at term according to the institutional guidelines and regulations.

### Patient recruitment and tissue sampling

Women undergoing elective caesarean section without uterine contractions formed the not-in-labour (N, n = 31) group. Women who entered spontaneous and established labour for at least 2 h, but required emergency surgery, formed the term in-labour (L, n = 29) group. Following delivery, the placenta and membranes were examined for chorioamnionitis. Women displaying clinical or histological indications of infection were excluded from the study. Clinical indications for the emergency section L group included breech, macrosomia, foetal distress, failure to progress and low amniotic fluid index, while subjects in the N group underwent surgery owing to previous caesarean sections. All women were between 36 completed weeks and 40 weeks gestational age. Tissues were sampled using a surgical technique standardized such that the bladder is reflected off the lower segment of the uterus (the uterovesical-fold) and the uterine incision is made in the upper part of the lower segment. Connective tissue and decidua were removed, and the tissues snap frozen in liquid nitrogen and stored at −80°C until analysis.

The tissues were extracted by the guanidinium thiocyanate-acid phenol-chloroform method as described previously (Chan *et al.*, 2002) and 21 genes experimentally found by the RNA subtraction method to be differentially up-regulated were analysed by Bioinformatics (Ingenuity Pathway Analysis) analyses and published data. Twelve genes identified from the post-subtraction *in silico* analyses were added to the earlier group of 21 genes for confirmation by quantitative RT-PCR, with only 29 of the 33 found to increase significantly with labour in a cohort separate from the subtraction study (Chan *et al.*, 2002; Supplementary Table SI). Z-scores for each gene were calculated from the data of all 60 women, and the z-scores of the 29 mRNA species for each mother were combined to form her overall gene expression ranking in the study cohort. For each mother, a radar plot was generated including data for the 29 genes. Mothers were then ranked from 1 to 60 based on their mean z-score data (Supplementary Fig. S1a). The radar plots were combined to generate a cigar plot showing the transition from not-in-labour to in-labour status, which largely, but not completely, aligned with the clinical characterization of their status (Supplementary Fig. S1b). This is consistent with uncertainty over how close to labour and delivery each mother may be, as clinical characterization is notoriously difficult for both the woman and her clinician (Romero *et al.*, 2006; Hanley *et al.*, 2016), while the cervical changes used to diagnose the mother’s labour status can be misleading since many patients with cervical shortening show no clinical signs of uterine contractions (Myers *et al.*, 2015). We propose that the ranking order represents a surrogate for time that indicates the position of the mother on the pathway to labour and delivery. Candidates for whole transcriptome analysis using RNAseq were chosen from the two distal ends of the cigar plot (Supplementary Fig. S1b, i.e. non-labouring (n = 6), and labouring (n = 6) groups as well as women clinically classified as labouring from the middle band to represent mothers in early labour (n = 5)).

### RNA sequencing

Paired-end sequencing of 17 human whole RNA stranded samples was performed on an Illumina HiSeq 2500 platform at the Australian Genomics Research Facility Ltd. (AGRF). Sample integrity was checked on an Agilent Bioanalyzer and all samples had RNA integrity number >7. Samples were first depleted of ribosomal RNA with Illumina Ribo-zero Gold. The rRNA-depleted samples were fragmented using heat and divalent cations before reverse transcription with SuperScript II kit (Invitrogen). Ultra-deep sequencing was carried out generating 100–150 million usable reads per sample. The whole RNA content therefore includes both protein-coding and ncRNA transcripts.

### Data analysis

#### RNAseq data analysis

Clean RNAseq reads were aligned to human genome build hg38 using the HISAT2 (Kim *et al.*, 2019) aligner. The aligned (.bam) files were then used to summarize counts for known protein-coding and non-coding gene annotations (GENCODE version 33) using the FeatureCounts utility of the Subread package (Liao *et al.*, 2019, 2014). During the quality control step, it was observed that non-labouring samples (except for EC1) clustered together but samples from the early and late labour group were not clearly separated in the hierarchical clustering (Supplementary Fig. S2a–c). Based on these observations, we first removed one sample (EC1) from the non-labouring group and the remaining 16 (n = 5 + 5 + 6) samples were further processed. Second, we identified a signature based on groups of protein-coding genes associated with each of the phenotypic groups (Supplementary Fig. S2d).

Coding-gene counts were used to identify ‘modules’ of co-expressed genes and generate gene expression signatures related to each sample within a phenotypic group (Zhang and Horvath, 2005). Thus, from the whole transcriptome data, we generated the gene expression signatures representative of each of the no-labour, early labour and late labour groups by considering co-expressed gene modules using an R-based tool called CEMiTool (Russo *et al.*, 2018). Gene set enrichment analysis (GSEA) was performed for each module. GSEA associated activity of each module within each sample class was then represented as a net enrichment score, which corresponds to a shifting of gene set constituents of a module towards either end of a ranked list representing strongly positive or negative correlations. To associate biological function to each of the modules, we examined over-represented pathways in each co-expressed module.

From the reads, two classes of ncRNA profiles were also generated, these were long non-coding RNA (lncRNA) and microRNA (miRNA) using GENCODE (Frankish *et al.*, 2019) and miRBASE (Kozomara *et al.*, 2019) reference annotations, respectively. FeatureCount (Liao *et al.*, 2014) and EdgeR-Voom packages (Robinson *et al.*, 2010; Law *et al.*, 2014) were used to perform the gene expression analysis. The ncRNA counts vary in their abundance and additionally, a co-expression network analysis was not possible for a large number of ncRNA captured in our analysis. Therefore, for ncRNA expression analysis, we combined the two labouring groups (n = 5 + 6) into one and compared this single group against the non-labouring group (n = 5). Since miRNAs are known to repress expression of their target mRNAs and have inverse correlations with their target lncRNAs, we computed negative Pearson correlations between the miRNA:mRNA and miRNA:lncRNA. We summarized results for adj.pval < 0.05, and at different correlation cut-offs. As lncRNA is known to negatively regulate miRNAs, we confirmed known miRNA:lncRNA and gene:target pairs using databases such as Starbase (Yang *et al.*, 2011) and RNAInter (Lin *et al.*, 2020) that contain experimental data from multi-omics studies.

Using the overrepresentation analysis tool from the Reactome database (Reimand *et al.*, 2019), we performed pathway enrichment analysis of differentially expressed miRNAs. This uses a hypergeometric distribution to test for significance and false discovery rate (FDR) to correct for multiple testing (Reimand *et al.*, 2019). Gene set analysis of lncRNA was performed using the lnCompare database (Carlevaro-Fita *et al.*, 2019), and genes miRNet2.0 (Chang *et al.*, 2020) for miRNA.

#### Regulatory elements

The 3-dimensional structure of the genome and chromatin remodelling facilitates transcription, and the compartmentalization of transcriptional activities can be deduced by aggregating various types of genomic and epigenomic data. We have used this combined information to suggest causal relationships leading to an expression phenotype.

##### Topologically associated domains

Chromosomal looping can bring arrays of regulatory elements from distant parts of the genome to create high-level self-interacting contacts and form sub-mega base-pair TADs (Lieberman-Aiden *et al.*, 2009; Bonev and Cavalli, 2016). Generally, TADs encompass interactions between enhancers and promoters, as well as between co-regulated genes, which reflect cell-type-restricted transcriptional programmes. Enhancers occur where arrays of permissive regulatory elements are grouped. TADs are surrounded by insulators at their boundaries, which prevent enhancers from exerting actions outside a domain (Zabidi *et al.*, 2015). It has been shown that constitutively expressed genes, such as housekeeping genes, tend to be located at the boundaries of TADs (Dixon *et al.*, 2012; Muro *et al.*, 2019). Genes with more specialized roles tend to concentrate within a domain and may be under tighter gene regulatory control. We mapped genes from the three modules to their genome-wide contact domain locations (Rao *et al.*, 2014).

##### Active enhancers

We investigated whether more than one of our genes of interest were located inside a TAD, and if they were sharing active enhancers. EnhancerAtlas 2.0 (Gao and Qian, 2020) was used to identify active enhancers associated with the genes that comprised the three identified gene modules. Next, we investigated the transcription factors (TFs) potentially involved in epigenome reprogramming for labour.

##### TF binding sites

To complete the regulatory complex view of the gene expression environment, we looked for TF binding sites (TFBS) both in the enhancer (EnhancerAltas classification) and promoter (10 000 bp upstream and 5000 bp downstream of TSS) regions using a list of known TF motifs from the JASPAR database (Sandelin *et al.*, 2004; Khan *et al.*, 2018). In order to identify significantly enriched TFs, we subtracted the occurrence of these motifs by chance to obtain statistically enriched TFBS. The results were selected using an adjusted *P-*value < 0.05 cut-off (Kwon *et al.*, 2012; Gearing *et al.*, 2019).

## Results

### Gene expression signatures and cellular functions

**Figure 2:**
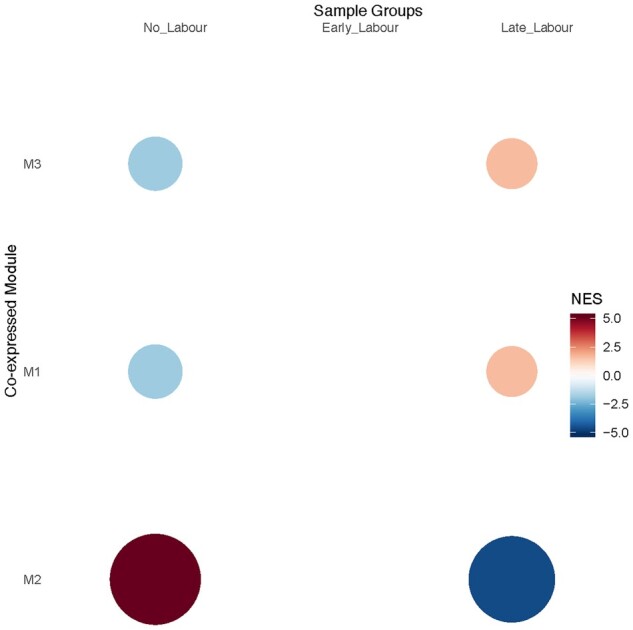
**Gene set enrichment analysis for myometrium of the human uterus.** The net enrichment score (NES) for three samples groups are plotted on the *x*-axis; NL, no labour; EL, early labour and LL, late labour. The red colour represents higher activity and blue represents lower activity. The size of the circle is proportional to the NES scores. The average expression of the genes from the three modules is provided in Supplementary Table SII.

The GSEA (Mootha *et al.*, 2003; Subramanian *et al.*, 2005) resulted in three significant modules represented as M1, M2 and M3 (Fig. 2) and were composed of 81, 46 and 36 genes, respectively. We observed that M2 had higher expression in the no-labour stage (red), while expression of M1 and M3 was associated with the active labour state (Fig. 2). We did not observe a co-expressed gene module that can characterize the early labour stage; this may be due to high variance in this group relating to the intermediate state of the members of this group on the trajectory to labour.

**Figure 3:**
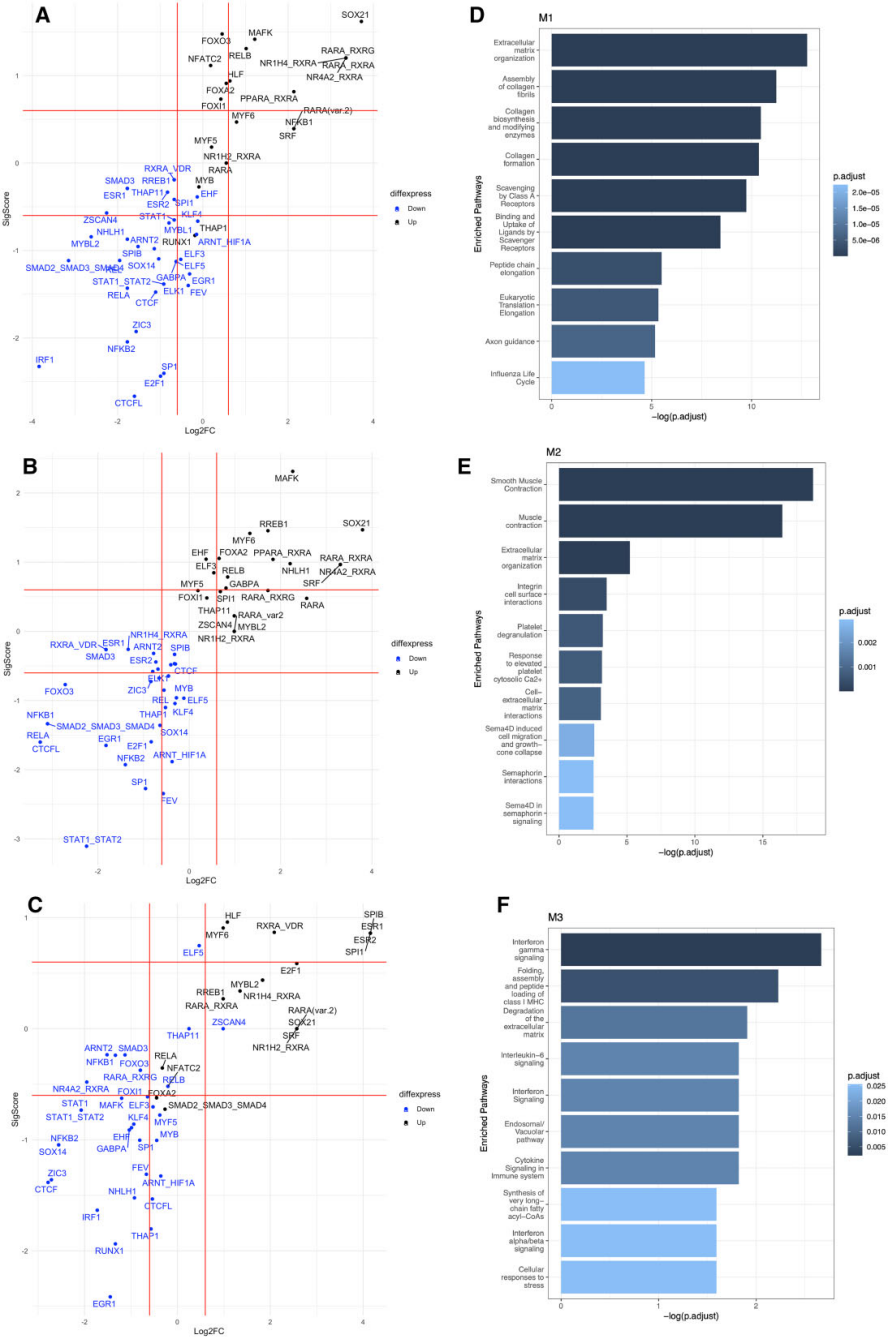
**Active enhancers and pathways analysis for myometrium of the human uterus.** The column on the left (**A–C**) shows transcription factor (TF) enrichment in the enhancers plotted as their log2 fold change between identified genes and the background sets versus the significance score computed as –(log2FC)*log10(*P*-value). TFs over- and under-represented for each of the three modules are shown in black and blue, respectively. The column on the right (**D–F**) shows pathway enrichment for genes in the modules M1, M2 and M3, respectively.

The M1 module is enriched for pathways associated with matrix degradation (Fig. 3A), the M2 module exhibits a smooth muscle profile (Fig. 3B), and the M3 module shows enrichment for immune gene signatures (Fig. 3C). The size and strong positive correlation of the M2 module with the no labour state suggests the base-level expression signature of myometrium, and that these genes are associated with the maintenance of pregnancy. Some of these observations, such as the expression of cytokines and immune pathways in the M1 and M3 modules, are consistent with previous studies of the gene signatures of spontaneous term labour (Bethin *et al.*, 2003; Keelan *et al.*, 2003; Bukowski *et al.*, 2006; Stanfield *et al.*, 2019). Average expression of genes from the three modules is provided in Supplementary Table SII.

### TADs may be related to co-regulation of gene expression

**Figure 4:**
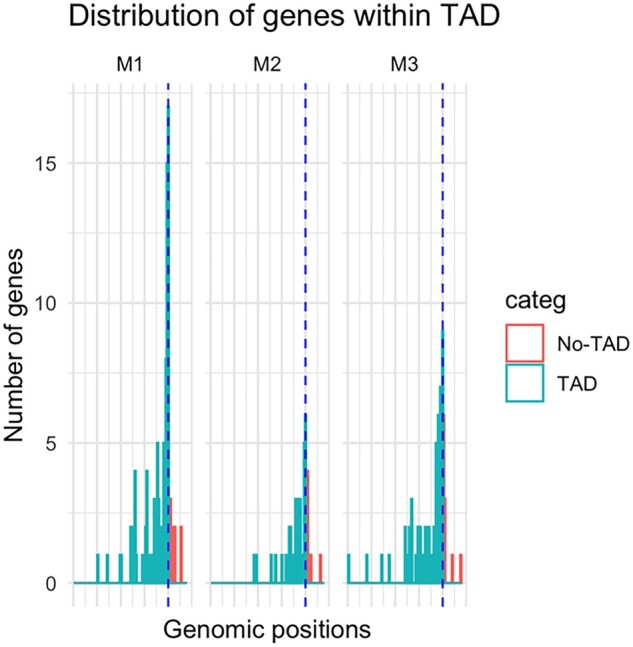
**Distances of genes from modules M1, M2 and M3 calculated from the nearest known topologically associated domain (TAD) boundary.** Here, the dashed blue line denotes the TAD boundary, the histogram of gene counts shows that the majority of the genes are inside known TADs and a small proportion lie near the boundary or outside the domain. As shown, 66% of M1 and M3 genes, and 76% of M2 genes lie within the TAD regions.

Mapping of our co-expressed gene modules to previously published genomic coordinates of TADs (Rao *et al.*, 2014) was compared with that of randomly selected genes. We found that the genes from the three modules are non-randomly distributed (Kolmogorov–Smirnov two-sided test *P*-value = 0.0006), with 76% of M2 genes, and 66% of both the M1, and M3 groups predominantly located within a topological domain as compared to randomly selected genes (Fig. 4). This suggests that the majority of the genes within the three co-expressed modules have specialized functions and are likely to be co-regulated. M1 genes at a TAD border are *COL1A2*, *IGFBP5*, *HSPA8*, *SPARCL1*, *COL5A1*, *ENO1*, *MACF1*, *GAPDH*, *CANX*, *VWF*, *FBLN1*, *UBC*, *HSP90AB1*, *RPL4*, *CSDE1*, *ALDOA*, *RACK1* and *RPS3*, which have roles in glycolysis, glucogenesis and amino acid biosynthetic pathways. Similarly, M2 genes at the border are *FLNA*, *MYH11*, *AHNAK*, *FLNC*, *CNN1* and *MAP1B*, which are involved in muscle fibre development and cell junction assembly. Finally, M3 genes at the border are *VCAN*, *MSN*, *MMP2*, *HLA-E*, *HLA-B*, *STAT3*, *VMP1*, *RDH10* and *CXCL8*, which have roles in cytokine signalling and immune pathways.

**Figure 5:**
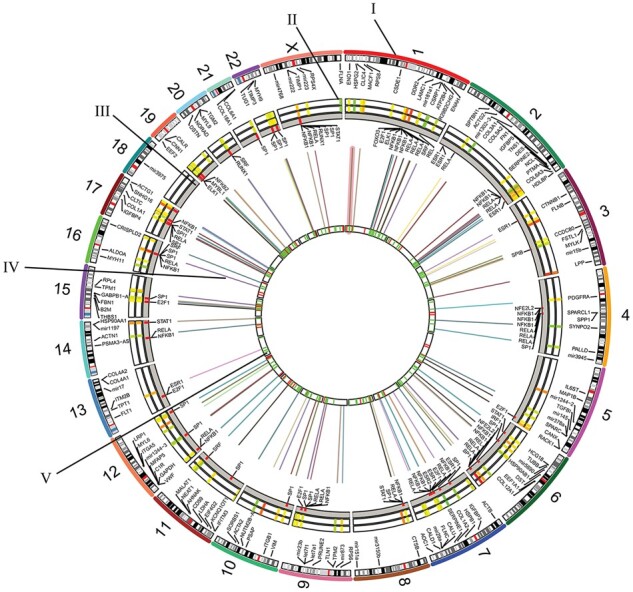
**Map of genomic and epigenomic factors driving the human term labour phenotype.** Outermost track (I) displays coding genes from the three identified modules, and non-coding genes (long non-coding RNA and microRNA: mir). Next, the gene expression heatmap (II) is plotted as up- (red) and down-regulated (yellow). This is followed by the transcription factor binding site track (III). Enhancer–promoter interactions are shown by multi-coloured lines (IV). The innermost circle (V) shows the TAD (green) or No-TAD (red) location of the genes in the outermost layer. TAD, topologically associated domain.

**Figure 6:**
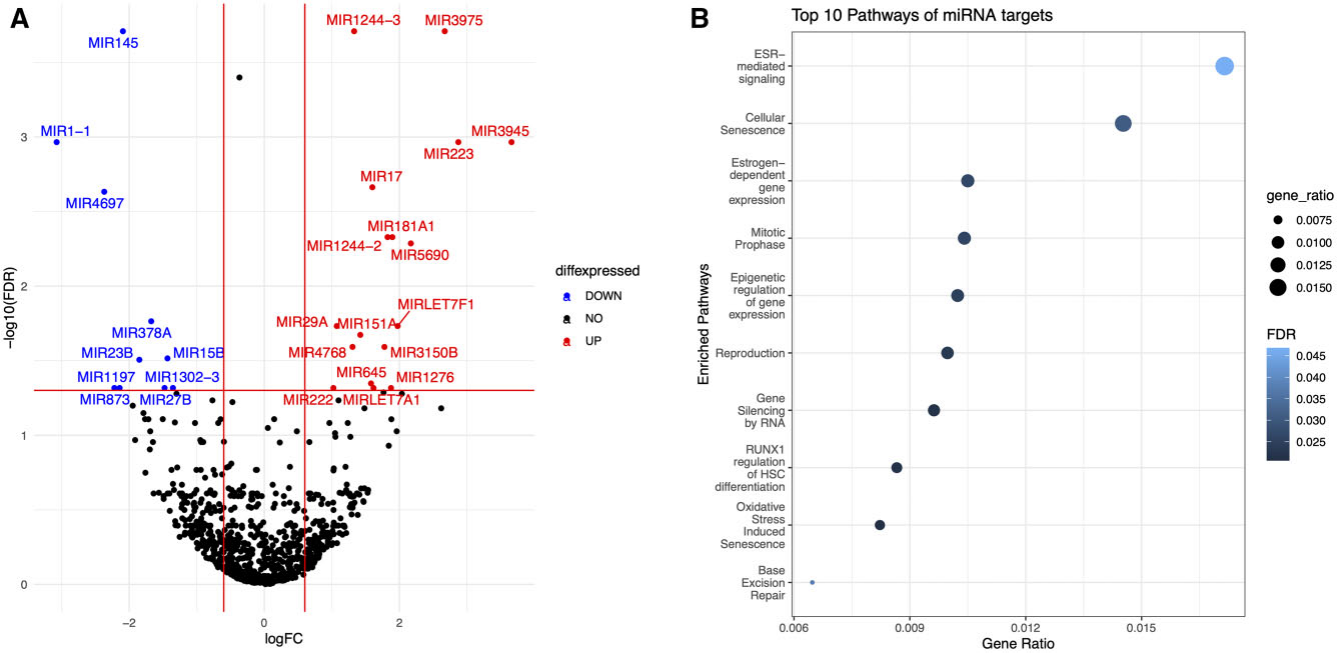
**Differential expression and pathway analysis of miRNA between labour and no-labour groups.** (**A**) Volcano plots of miRNA differential expression between no-labouring and labouring (early labour + late term labour combined) groups. The *X*-axis shows log2 of fold change (FC) between the two conditions, and *Y*-axis plots negative log10 of adjusted *P*-value. The gene with a fold change of 1.5 and false discovery rate (FDR) ≤ 0.05 are highlighted as red (up) or blue (down). Genes with unchanged expressions are shown as black dots. (**B**) Top 10 enriched pathways from Reactome overrepresentation analysis of miRNA: histone modification interactions, with ranking based on the gene ratio of input genes relative to background and adj. *P*-value ≤0.05 indicated by the blue colour bar gradient. The size of the bubbles also reflects the magnitude of the gene ratio. The figure was produced using the R package ‘ggplot’. The data emphasize the importance of estrogen receptor (ESR)-mediated signalling. miRNA, microRNA.

TADs can be subdivided into subdomains that contain genes that have a higher likelihood of co-regulation. Enhancer–promoter interactions are required to form regulatory units and co-regulate genes in these subdomains. We mapped enhancer–promoter interactions onto our gene modules. These enhancer–promoter interactions are marked as red links in Fig. 5. Furthermore, analysis of TFBS in the promoter and enhancer regions revealed TFs that may regulate these genes, creating transcriptional units. We have demonstrated over- and under-represented TFs in the active enhancers for the three different gene modules (Fig. 6A–C). All three modules shared nine common TFs (NR1H2::RXRA, MYF6, RARA::RXRA, PPARA::RXRA, RARA(var.2), FOXA2, SRF, RARA, SOX21) in their enhancers. Five TFs were specific to M1 (THAP1, NFKB1, MYB, FOXO3 and RUNX1), six TFs were specifically found in the enhancer regions of M2 (EHF, NHLH1, ELF3, GABPA, THAP11 and ZSCAN4) and 10 were M3 specific (RXRA::VDR, ESR1, ESR2, SPIB, E2F1, ELK1, REL, MYBL1, RELA and SMAD2::SMAD3::SMAD4). Overrepresented TFBS restricted to the promoter regions are summarized in Table I and as shown in Supplementary Fig. S3, the predictive scores of these TFBS are not affected by the genomic GC content.

**Table I:** Transcription factor enrichment in promoter regions, 1000 bp upstream and 5000 bp downstream of transcription start site.

Module	Transcription factor	JASPAR ID	Family	Z-score
M1	Klf4	MA0039.2	BetaBetaAlpha-zinc finger	19.788
	ELF5	MA0136.1	Ets	14.911
	SPIB	MA0081.1	Ets	14.88
	NFATC2	MA0152.1	Rel	14.189
	CTCF	MA0139.1	BetaBetaAlpha-zinc finger	14.041
	RREB1	MA0073.1	BetaBetaAlpha-zinc finger	12.698
	Egr1	MA0162.1	BetaBetaAlpha-zinc finger	12.294
	AP1	MA0099.2	Leucine Zipper	11.918
	SP1	MA0079.2	BetaBetaAlpha-zinc finger	11.897
	REL	MA0101.1	Rel	11.878
	FEV	MA0156.1	Ets	10.903
	RUNX1	MA0002.2	Runt	10.883
	SPI1	MA0080.2	Ets	10.316
	SRF	MA0083.1	MADS	10.171
M2	SRF	MA0083.1	MADS	33.716
	ELF5	MA0136.1	Ets	25.815
	SPIB	MA0081.1	Ets	25.182
	RUNX1	MA0002.2	Runt	21.96
	TEAD1	MA0090.1	Homeo	16.75
	PPARG::RXRA	MA0065.2	Hormone-nuclear Receptor	16.15
	NFATC2	MA0152.1	Rel	15.48
	Hand1::Tcfe2a	MA0092.1	Helix-Loop-Helix	15.36
	REL	MA0101.1	Rel	14.84
	FEV	MA0156.1	Ets	14.80
	Foxa2	MA0047.2	Winged Helix-Turn-Helix	14.77
	TBP	MA0108.2	Beta-sheet	14.08
	MEF2A	MA0052.1	Other Alpha-Helix	14.03
	Klf4	MA0039.2	Zinc-coordinating	13.55
	SOX9	MA0077.1	Other Alpha-Helix	12.92
	MEF2A	MA0052.1	MADS	12.85
	AP1	MA0099.2	Leucine Zipper	12.75
	HOXA5	MA0158.1	Homeo	11.96
	MYB	MA0100.1	Myb	11.86
M3	STAT1	MA0137.2	Stat	22.02
	Gata1	MA0035.2	GATA	17.58
	NF-kappaB	MA0061.1	Rel	17.53
	RELA	MA0107.1	Rel	17.25
	CTCF	MA0139.1	BetaBetaAlpha-zinc finger	16.78
	FEV	MA0156.1	Ets	11.24
	FOXI1	MA0042.1	Forkhead	10.51

Over-representation of transcription factor binding sites from the JASPAR database was analysed. The top results were ranked by Z-score at a cut-off of Z-score = 10.

### ncRNA mediated regulation revealed by analysing gene expression profiles and target analysis

#### miRNA expression profiles

For profiling ncRNA expression, we merged the early and late labour groups and tested for differential expression between the no-labour and labour groups. The expression of 27 miRNAs changed significantly (FDR adjusted *P*-value ≤ 0.05) in the labour groups (Fig. 6A). The expression of 10 miRNAs decreased from the no-labour to the labour groups, whereas, 17 miRNAs increased their expression in the labour groups. More detailed annotations of these miRNA are available in Supplementary Table SIII.

miRNA targets, namely mRNA and lncRNA, were identified and then analysed for their involvement in different biological pathways. As shown in Fig. 6B, a total of 25 enriched pathways (adj. *P*-value ≤ 0.05) were found for histone modification targets of these miRNAs. Several relevant pathways, such as estrogen receptor (ESR)-mediated signalling, estrogen-dependent gene expression, reproduction and oxidative stress induced senescence, showed high levels of enrichment.

We observed that *hsa-miR-223*, *hsa-miR-let-7a* and *miR-145* were highly expressed in the no-labour and low in the labour group. *hsa-miR145* was at the top of the significantly differentially expressed list with more than 4-fold down-regulation in labour samples as compared to the no-labour group. *hsa-miR145* was found to be negatively correlated with ESR1 expression, and several other coding genes. In breast cancer, *hsa-miR-145* has been confirmed to be involved in pro-apoptotic activities in collaboration with TP53 and represses expression of ESR-alpha by directly binding to its 3′UTR (Spizzo *et al.*, 2010). The data also included two other miRNAs (*hsa-miR-181a-d* and *hsa-miR-222*) known to be involved in ESR1 pathways (Mou *et al.*, 2017). *mir-181a* and *hsa-miR-222* were low in the no-labour and high in the labour groups. Previously, *miR-181a*, *hsa-miR_23a* and *hsa-miR-26b* were also shown to directly regulate progesterone receptors (Gilam *et al.*, 2017). The data also include four miRNA (*hsa-let-7a-1*, *hsa-let-7f-1*, *hsa-mir-223* and *hsa-mir-29a*) that are known targets of NFkB (Kumar *et al.*, 2014; Liu *et al.*, 2015b). Among the other differentially changed miRNA, *hsa-miR-132* and *hsa-miR-133* are known to regulate estradiol synthesis (Dai *et al.*, 2013; Li *et al.*, 2015; Wu *et al.*, 2015); *hsa-miR-223*, *hsa-miR-132*, *hsa-miR-199a* and *hsa-miR-31* have a regulatory role in chromatin remodelling (Aprelikova *et al.*, 2010; Alvarez-Saavedra *et al.*, 2011; Sakurai *et al.*, 2011; Zardo *et al.*, 2012; Pagano *et al.*, 2013). Additionally, *hsa-miR-222*, *hsa-miR-141*, *hsa-miR-146*, *hsa-miR-146a*, *has-miR-214*, *hsa-miR-33a* and *hsa-miR-411* regulate oxidative stress (Wommack *et al.*, 2018). We performed a correlation analysis between these 27 miRNAs and their 5235 identified mRNA targets. Out of these correlated miRNA:mRNA pairs 48.95% were negative correlations, of which 11.95% were statistically significant (adj. *P*-value < 0.01; Supplementary Table SIV).

A pathway enrichment of Kyoto Encyclopedia of Genes and Genomes (KEGG) biological network of miRNA:mRNA targets (Pearson correlation > 0.8 and adj. *P*-value <0.05) from our RNAseq data is also drawn in Supplementary Fig. S4. Genes in these networks are enriched for NFkB signalling, innate immune and cytokine pathways. Protein domain analysis showed enrichment for histone (H4/H2B) and zinc-binding domains, SNT, Cullin and RNA-binding domains, as confirmed with SMART (Letunic *et al.*, 2021) and InterPro database (Blum *et al.*, 2021) analysis. These proteins were enriched for splicing and RNA transport gene ontologies.

#### lncRNA expression profiling

**Figure 7:**
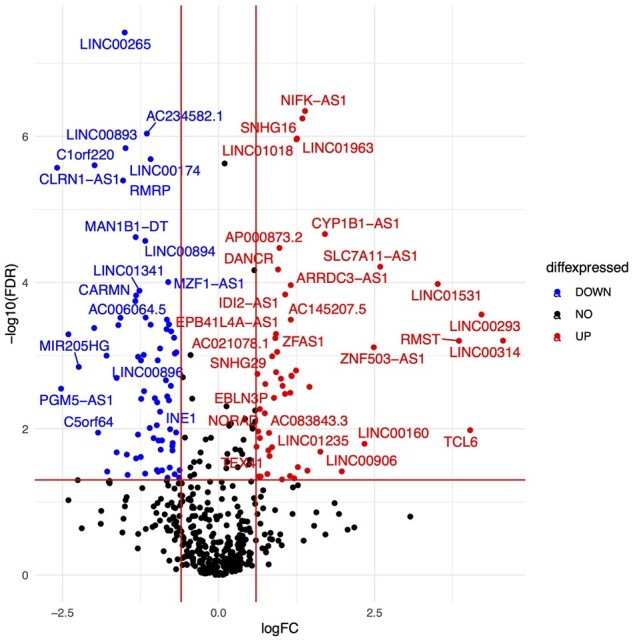
**Volcano plots of long non-coding RNA (lncRNA) differential expression between labour and no-labour groups.** Plots are shown for the no-labouring and labouring (early labour + late term labour combined) groups. The *X*-axis shows log2 fold change between the two conditions, and *Y*-axis plots the negative log10 of the adjusted *P*-value. The gene with a fold change of 1.5 and false discovery rate (FDR) ≤ 0.05 are highlighted as red (up) or blue (down). lncRNA with unchanged expression are shown as black dots.

In the differential expression analysis of the transcriptome, we identified 146 differentially changed lncRNA (FDR ≤ 0.05) that are known targets of miRNA differentially expressed in our analysis (Fig. 7). Of these, two lncRNA (AK054607 or SOCS2-AS1, and LINC00312) have previously been reported in spontaneous labour transcript profiles (Romero *et al.*, 2014). When compared with the background set of lncRNA, mean expression of these lncRNAs at term was found to be higher than expected by chance in all groups.

Further, we looked at the distribution of the altered lncRNAs with respect to various genomic features. We observed that the majority of the lncRNA were in antisense and divergent orientation relative to their nearest coding genes, and these lncRNAs are preferentially distributed around their closest coding genes (Supplementary Fig. S5). Eighty-three of these lncRNAs have been previously reported in association with disease in the databases lncRNA disease (Chen and Yan, 2013), lnc2cancer (Ning *et al.*, 2016) and CLC (Carlevaro-Fita *et al.*, 2017). Another general observation was that their spliced length was longer, and conservation in 20 mammals was higher than expected (data not shown). More investigation would be required to assess whether these properties have functional consequences.

The distribution of these lncRNA suggests that they may be acting as co-regulators to the closely co-located protein-coding genes. These associations can be either positive or negative resulting in enhanced expression or repression of the adjacent protein-coding gene. To test the co-differential expression of mRNA and lncRNA, a correlation analysis between lncRNA:mRNA, and lncRNA:miRNA was performed. There were 2602 mRNA as common targets for both miRNA and lncRNA with a significant negative correlation between miRNA:lncRNA (adj. *P*-value < 0.01; Supplementary Table SIV and Fig. S4). The gene ontology suggested that these common targets are involved in RNA processing, splicing, oxidative stress and epigenetic gene regulation processes.

### Integrated pathway analysis reveals a combinatorial regulatory network of genes, TFs and ncRNA

We used genes from the three co-expressed modules, their identified TFs, differentially expressed miRNA and their lncRNA targets to perform integrative pathway analysis.

**Figure 8:**
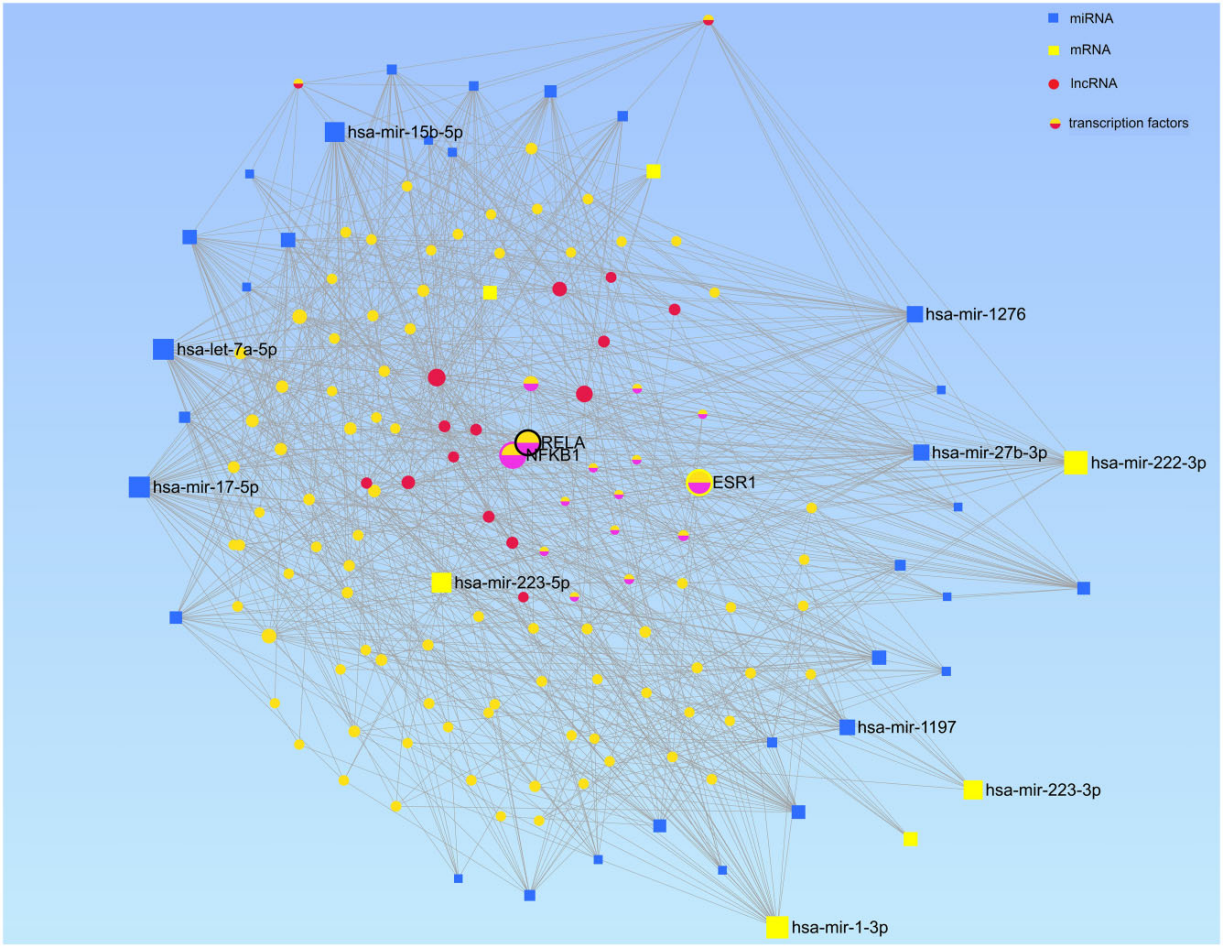
**Integrated co-regulatory network analysis.** The gene-target interaction network of mRNA (yellow), miRNA (blue), lncRNA (red) and transcription factors (pink) obtained from our co-expression analysis (false discovery rate (FDR) ≤ 0.05). The network reveals important hub nodes shown as yellow highlights. The size of the node corresponds to enrichment in top pathways. lncRNA, long non-coding RNA; miRNA, microRNA.

The nodes represent an mRNA (yellow), miRNA (blue), lncRNA (red) or a TF (pink), and the nodes are ranked by their degree and betweenness (Fig. 8). Degree denotes the number of edges connected to a node, while betweenness is a measure of how central a node is in the network. Nodes with high betweenness essentially serve as bridges between different portions of the network i.e. interactions must pass through this node to reach other portions of the network.

Fifteen lncRNA (MCM3AP-AS1, TUG1, MIR29B2CHG, HCG18, LINC00963, KCNQ1OT1, NEAT1, HELLPAR, SNHG16, NUTM2B-AS1, MALAT1, PSMA3-AS1, GABPB1-AS1, NORAD and NKILA), and three TFs (ESR1, RELA and NFkB1) showed high degree (≥50), and hence acted as hubs in the network. The following 15 TFs were the most highly overrepresented: NFKB1, SP1, RELA, ESR1, STAT1, E2F1, RUNX1, REL, IRF1, SRF, ESR2, NFE2L2, ELK1, SPI1 and FOXO3.

From our list of 27 differentially expressed miRNA, 21 showed high degree (>10) in the network (*hsa-let-7a*, *hsa-let-7f*, *hsa-mir-1*, *hsa-mir-1197*, *hsa-mir-1276*, *hsa-mir-145*, *hsa-mir-151a*, *hsa-mir-15b*, *hsa-mir-17*, *hsa-mir-181a*, *hsa-mir-222*, *hsa-mir-223*, *hsa-mir-23b*, *hsa-mir-27b*, *hsa-mir-29a*, *hsa-mir-3150b*, *hsa-mir-378a*, *hsa-mir-3975*, *hsa-mir-4768*, *hsa-mir-5690* and *hsa-mir-873*). These miRNAs appear to be regulated by the previously mentioned 15 different TFs (Supplementary Table SIII).

## Discussion

Understanding complex interactions between different functional genomic factors driving a phenotype requires integrated analysis of high-throughput genomics datasets. Here, we provide a workflow combining existing and new omics data to dissect the role of different omics layers in regulating term labour. Our approach provides a holistic view of the genome architecture and combinatorial interactions within the various functional layers that bring about changes in the muscle of the uterus at term labour (Fig. 1).

To the best of our knowledge, no previous RNAseq studies have sequenced the whole transcriptome of myometrium at the ultra-deep level (>100 million reads/sample). We obtained co-expressed gene modules and ncRNA expression profiles associated with non-labouring and labouring groups. We also identified evidence for the active enhancer–promoter pairs and the TFs that directly regulate the gene modules corresponding to labouring and non-labouring groups.

A number of lncRNA changed significantly in term labour samples (Ramírez-Colmenero *et al.*, 2020). We discuss, as an example, lncRNA NKILA that is known to directly interact with and inhibit NFkB pathways by binding to P65 (Liu *et al.*, 2015a; Huang *et al.*, 2016, 2018). It has been shown previously that placental production of corticotropin-releasing hormone (CRH) is linked to gestational length in human, with a more rapid increase in CRH linked to preterm birth (McLean *et al.*, 1995; Smith, 2015). CRH is also linked to the exponential rise in maternal oestriol in late pregnancy (Ellis *et al.*, 2002), and oestriol dominance over estradiol to the onset of preterm and term labour (Smith *et al.*, 2013, 2009). CRH has also been shown to stimulate the NFkB system, which can initiate labour (Zbytek *et al.*, 2004). There is strong evidence that estrogens and NFkB are key components in the onset of labour. Progesterone withdrawal during labour up-regulates ESR1 and allows estrogen action (Mesiano *et al.*, 2002). The ESRs can activate target genes either through direct binding to an estrogen-responsive element in the target gene promoter, or indirectly through interaction with another DNA-binding protein such as NFkB. We found the expression of lnc species NKILA down from control to the early labour group and up again in the late labour. This is consistent with NKILA preventing the onset of labour by suppression of NFkB pathways during pregnancy but a fall of NKILA allowing the onset and progression of labour via NFkB activation. Integrative analysis revealed 14 new lncRNAs as hub genes in a combinatorial regulatory network. Our data indicate that the lncRNA target their nearest coding genes in a sense-antisense manner.

There is a small but growing body of research that suggests that miRNA can be involved in regulating molecular mechanisms underpinning uterine muscle contraction during term labour (Elovitz *et al.*, 2014; Ackerman *et al.*, 2018; Cook *et al.*, 2019). miRNA activity can be impacted by interactions with other molecules. For example, the ‘sponge effect’ refers to an interaction of miRNA with lncRNA. The lncRNA usually has a fully complementary sequence that matches a given miRNA and is thus able to bind to that miRNA, preventing it from inhibiting mRNA translation. An lncRNA that acts in this manner has been named as a competing endogenous RNA (Salmena *et al.*, 2011). We provide evidence that miRNA and lncRNA work synergistically in the form of complex networks to regulate expression of their target genes in term labour. We selected lncRNA:miRNA pairs that have previously been confirmed by CLIP-Seq (Hafner *et al.*, 2021) captures.

The integrative network analysis showed key enriched pathways regulated by ESR1, NFkB, RELA and SP1 TFs. Our data showed that these ncRNAs and TFs are involved in regulating estrogen action, oxidative stress, histone modification and chromatin modelling. These network signals are therefore strongly associated with pathways that bring about changes in genome conformation resulting in cascading transcriptional changes leading to the labour phenotype.

Our analysis of TFBS in the promoter and active enhancers identifies TFs responsible for driving module-specific co-regulation. As a result, we report three groups of TFs regulating co-expression of the smooth muscle phenotype in M2, and those driving transcription of modules enriched in labour specific modules M1 and M3. As expected, binding sites for TFs RELA, NFkB and ESR1 were found overrepresented in the regulatory regions of M1 and M3 genes only, and not for the M2 genes.

## Conclusion

Collectively, our results demonstrate the epigenomic signatures, and transcriptional responses associated with a term labour phenotype. We identified key components of a likely complex regulatory network that works through combinatorial interactions to drive these changes. Pregnancy hormones at term will induce a cascade of signalling pathways that will in turn affect chromatin conformation, thereby exposing regulating elements, and biomolecule interactions to carry out large-scale transcriptional changes within TADs; as a result the myometrium changes from a relaxed to a contractile phenotype.

Ultra-deep sequencing of the whole transcriptome made it possible to gain a global profile of the term labour transcriptome at high resolution. ncRNAs vary hugely in their size and abundance and analysing short and long ncRNA together increased the sensitivity of analysis. The abundance of differentially expressed ncRNAs necessitated a targeted analysis of different classes of ncRNA. Further, our gene profile signatures along with existing genome-wide chromatin capture data indicate that the 3-dimensional structure of the genome can determine the formation of specific transcriptional units. Our work informs future chromatin conformation captures from term non-labour and labour cohorts to dissect corresponding chromatin domains and subdomains.

Overall, we demonstrate the power of integrated analysis to obtain a holistic view of the term labour phenotype. Such approaches will expedite discovery of robust and reproducible treatments for pregnancy complications related to labour, such as preterm birth, dystocia and postpartum haemorrhage.

## Supplementary data

Supplementary data are available at *Molecular Human Reproduction* online.

## Data availability

The authors confirm that all relevant data are included in the paper and/or its supplementary information files. Raw and some processed data have been uploaded to Gene Expression Omnibus (GEO) under the project ID GSE186763.

## Supplementary Material

gaac003_Supplementary_Data
