# Supplementary material for: Transcriptomic analysis reveals myometrial topologically associated domains linked to the onset of human term labour
Source: Mol Hum Reprod. 2022 Feb 12;28(3):gaac003. doi: 10.1093/molehr/gaac003 (PMC8903000; doi:10.1093/molehr/gaac003)
Supplement: gaac003_Supplementary_Data [file gaac003_supplementary_data.zip › Supplementary data final.pdf]

Transcriptomic Analysis Reveals Myometrial Topologically Associated Domains linked to Onset of Human Term Labor

Sonika Tyagi[1], Eng-Cheng Chan[2], Daniel BARKER[3], Patrick McELDUFF[3], Kelly A Taylor[2], Carlos Riveros[3], Esha Singh[4], Roger Smith[2,3]

[1]Central Clinical School, Monash University and the Alfred Hospital, Melbourne VIC Australia
[2]Mothers and Babies Research Centre, HMRI University of Newcastle, NSW Australia
[3]University of Newcastle, Newcastle, NSW Australia
[4] Department of Biotechnology and Biochemical Engineering, Indian Institute of Technology, New Delhi India

Supplementary data

Content:

Table SI Genes used to rank the myometrial samples

Table SII:
Average expression of the genes from three modules

Table SIII: Complete annotations of differentially expressed miRNAs

Table SIV:
miRNA:mRNA and miRNA:lncRNA target correlations. (separate excel sheet file)

Figure S1 a): Radar Plots of 60 women recruited in the study.

Figure S1 b): 3D Cigar Plots of ranked genes.

Figure S2: Hierarchical clustering of genes based on their expression profiles

Figure S3: TFBS predicted scores trends

Figure S4: The KEGG pathway enrichment for all miRNA targets

Figure S5-a) Gene Set Enrichment analysis of lncRNA

Figure S5-b): Common mRNA targets of miRNA and lncRNA

Table SI Genes used to rank the myometrial samples

Abbreviation	Gene Name	GB Accession No.
APP	Amyloid beta (A4) precursor protein	NM000484
CRSP6	Cofactor Required for SP1 transcriptional activation, subunit 6 77kD, Mediator complex subunit 17 MED17	NM004268; AF104254
CX43	Connexin 43; Gap Junction Protein Alpha-1	NM000165
ELAVL1	ELAV like RNA binding protein 1 (Embryonic lethal, abnormal vision, Drosophila-like 1), Hu antigen R, HuR	NM001419
ESR1	Estrogen Receptor 1; ERα	NM000125
GAPDH	Glyceraldehyde-3-phosphate dehydrogenase	NM002046
IFITM2	Interferon-induced transmembrane Protein 2	NM006435
IL8	Chemokine (C-X-C motif) ligand 8, CXCL, Interleukin 8	NM000584
KIAA	KIAA 0062, Solute Carrier Family 39 Member 14 SLC39A14; Zinc/metal ion transporter	XM046677
MMP2	Matrix Metalloproteinase 2; MMP2	NM004530
MMP3	Matrix Metalloproteinase 3; MMP3	NM002422
MMP9	Matrix Metalloproteinase 9; MMP9	NM004994
NAPG	NSF attachment protein gamma,N-ethylmaleimide-sensitive factor attachment protein, gamma	NM003826
NCOR1	Nuclear Receptor Corepressor 1, aka PPP1R109, TRAC1	NM006311
NCOR2	Nucelar Receptor Corepressor 1	NM006312
NFκB1	Nuclear Factor of Kappa light polypeptide gene enhancer in B-cells 1, p50	NM003998
NPM1	Nucleophosmin/Nucleoplasmin Family, Member 1	NM002520
PGCP	Plasma glutamate carboxypeptidase	NM016134
PRB	Progesterone receptor isoform B	NM000926
PRT	Progesterone receptor Total	NM000926
PTGER1	Prostaglandin E receptor 1 (subtype EP1), 42kDa	NM000955
PTGER2	Prostaglandin E receptor 2 (subtype EP2), 53kDa	NM000956
PTGFR	Prostaglandin F Receptor, FP	NM000959
PTGS2	Prostaglandin-Endoperoxide Synthase 2; COX2	NM000963
RELA	V-REL Avian Reticuloendotheliosis Viral Oncogene Homolog A, p65	NM021975
SERPINF1	Serpin peptidase inhibitor, clade F (alpha-2 antiplasmin pigment epithelium derived factor), member 1, PEDF, PIG35	NM002615
SOD2	Superoxide dismutase 2, mitochondrial; MnSOD	NM000636
TGFB1	Transforming growth factor, beta 1	NM000660
TPI1	Triosephosphate Isomerase 1	NM000365
UBC	Ubiquitin C	NM021009

Table SII: Average expression of the genes from three modules namely, M1, M2, and M3

GeneName	Ensembl Gene.ID	Average Expression
AOC1	ENSG00000002726	3.683010529
CXCL8	ENSG00000169429	8.502826181
MT2A	ENSG00000125148	10.09824479
NAMPT	ENSG00000105835	10.96241633
S100A9	ENSG00000163220	10.00302129
SOD2	ENSG00000112096	13.48975869
ADAMTS4	ENSG00000158859	10.41938089
CCDC71L	ENSG00000253276	9.195035674
DES	ENSG00000175084	13.91449474
ADAMTS9	ENSG00000163638	10.51795451

TFRC	ENSG00000072274	10.10201241
RDH10	ENSG00000121039	9.787402728
ACSL4	ENSG00000068366	9.71639804
SPP1	ENSG00000118785	9.732291532
SOCS3	ENSG00000184557	10.05671043
HSPB1	ENSG00000106211	11.04878878
SRGN	ENSG00000122862	10.01816618
CNN1	ENSG00000130176	12.00214273
SLC39A14	ENSG00000104635	10.88645822
IGFBP3	ENSG00000146674	10.3980987
MEDAG	ENSG00000102802	10.010869
HIF1A	ENSG00000100644	10.69219931
ACTG2	ENSG00000163017	13.69049279
SYNPO2	ENSG00000172403	11.98819625
LCP1	ENSG00000136167	10.1686467
FLNC	ENSG00000128591	12.05214629
SERPINE2	ENSG00000135919	10.86446415
SERPINE1	ENSG00000106366	11.97028646
FN1	ENSG00000115414	14.47931809
ACSL1	ENSG00000151726	10.12035664
VCAN	ENSG00000038427	12.79419011
TIMP1	ENSG00000102265	12.39453465
FLT1	ENSG00000102755	9.468737965
NCL	ENSG00000115053	10.34376265
CD93	ENSG00000125810	10.68993576
CSRP1	ENSG00000159176	12.6803365
IGFBP5	ENSG00000115461	12.76307763
HSP90B1	ENSG00000166598	11.2279051
MCL1	ENSG00000143384	11.02366369
IL6ST	ENSG00000134352	11.05436309
ACTA2	ENSG00000107796	12.77678069
GLUL	ENSG00000135821	11.84022408
MYL9	ENSG00000101335	11.98741536
MYLK	ENSG00000065534	12.26104065
TPM2	ENSG00000198467	12.99096266
PRUNE2	ENSG00000106772	11.05872237
COL1A1	ENSG00000108821	14.18000367
EPAS1	ENSG00000116016	10.77209
AKAP12	ENSG00000131016	11.21735674
ATP2B4	ENSG00000058668	11.4243529
TNS1	ENSG00000079308	12.27034044
VMP1	ENSG00000062716	10.74370608
HSP90AA1	ENSG00000080824	10.56634832
CRISPLD2	ENSG00000103196	12.11912255

MYH11	ENSG00000133392	14.01810802
ENAH	ENSG00000154380	9.793055114
SORBS1	ENSG00000095637	10.91718472
HSP90AB1	ENSG00000096384	11.5037444
ENO1	ENSG00000074800	12.16144954
HSPG2	ENSG00000142798	12.75435669
COL18A1	ENSG00000182871	11.54789372
PALLD	ENSG00000129116	11.34208842
DST	ENSG00000151914	10.91934664
DUSP1	ENSG00000120129	10.68477153
GREB1	ENSG00000196208	11.03526967
MAP1B	ENSG00000131711	10.98216914
THBS1	ENSG00000137801	14.92670038
FLNA	ENSG00000196924	15.46424945
PTMA	ENSG00000187514	10.72064919
TPT1	ENSG00000133112	11.4554499
CTGF	ENSG00000118523	10.91275137
MMP2	ENSG00000087245	11.44395303
LDHA	ENSG00000134333	11.71176962
COL5A2	ENSG00000204262	11.94859595
CANX	ENSG00000127022	11.77574717
COL3A1	ENSG00000168542	15.41363628
TNC	ENSG00000041982	14.21617871
AHNAK	ENSG00000124942	13.9605718
DDR2	ENSG00000162733	10.88806895
CCDC80	ENSG00000091986	10.96943469
COL6A3	ENSG00000163359	14.10754761
RPS8	ENSG00000142937	10.69341315
CALD1	ENSG00000122786	12.41714624
TAGLN	ENSG00000149591	13.21024867
HSPA5	ENSG00000044574	10.97911747
CD59	ENSG00000085063	11.16974582
ITGA5	ENSG00000161638	12.87587206
MSN	ENSG00000147065	11.7141508
COL1A2	ENSG00000164692	14.60149417
TGM2	ENSG00000198959	13.80361055
SERPINH1	ENSG00000149257	11.0081439
CTNNB1	ENSG00000168036	11.28788506
MFAP5	ENSG00000197614	10.87115731
RPLP0	ENSG00000089157	11.09364472
RHOB	ENSG00000143878	11.23689222
IFITM3	ENSG00000142089	11.04166428
STAT3	ENSG00000168610	11.09307014
DCN	ENSG00000011465	12.24982586

ITGB1	ENSG00000150093	12.05790769
LPP	ENSG00000145012	12.8680621
TPM1	ENSG00000140416	11.96892663
RPL4	ENSG00000174444	11.56844129
HLA-B	ENSG00000234745	11.32723207
ITM2B	ENSG00000136156	10.43522076
GAPDH	ENSG00000111640	12.14635215
CTSB	ENSG00000164733	12.56422294
PDGFRA	ENSG00000134853	10.9552357
COL12A1	ENSG00000111799	12.01256255
COL5A1	ENSG00000130635	12.46737682
CLIC4	ENSG00000169504	12.02884331
RPS3	ENSG00000149273	10.71917469
FTL	ENSG00000087086	12.38266321
CD44	ENSG00000026508	11.3374534
B2M	ENSG00000166710	12.12167305
CSDE1	ENSG00000009307	11.39549808
HLA-E	ENSG00000204592	11.34689083
TXNIP	ENSG00000265972	12.57939467
RPS6	ENSG00000137154	11.11900037
FBLN1	ENSG00000077942	11.7848271
HSPA8	ENSG00000109971	12.71919503
TUBB	ENSG00000196230	10.84292809
FLNB	ENSG00000136068	11.10051746
LRP1	ENSG00000123384	12.47661695
PTRF	ENSG00000177469	12.21986234
RACK1	ENSG00000204628	11.21276728
ACTG1	ENSG00000184009	13.11687198
IGFBP4	ENSG00000141753	12.79678861
TIMP3	ENSG00000100234	13.82411377
CALU	ENSG00000128595	11.23231903
EIF4G2	ENSG00000110321	11.43992613
SPARC	ENSG00000113140	13.91058555
HDLBP	ENSG00000115677	11.60264525
ALDOA	ENSG00000149925	11.37091664
C1R	ENSG00000159403	11.08087468
ACTB	ENSG00000075624	14.96246545
COL6A1	ENSG00000142156	12.63830998
PKM	ENSG00000067225	11.43603096
CALR	ENSG00000179218	11.09874273
RPL3	ENSG00000100316	11.41196941
EEF2	ENSG00000167658	13.12836625
VWF	ENSG00000110799	11.86358033
COL4A1	ENSG00000187498	13.78967137

CLTC	ENSG00000141367	11.19911967
EEF1A1	ENSG00000156508	13.30647082
RPS4X	ENSG00000198034	11.3715664
TPM4	ENSG00000167460	11.87664053
FBN1	ENSG00000166147	12.68454475
VIM	ENSG00000026025	11.32157334
FSTL1	ENSG00000163430	12.53274175
ACTN1	ENSG00000072110	12.01572356
TLN1	ENSG00000137076	12.64464275
MYH9	ENSG00000100345	13.20884494
PSAP	ENSG00000197746	12.64303252
TGFBI	ENSG00000120708	12.01132309
MACF1	ENSG00000127603	12.26709091
MYL6	ENSG00000092841	11.59150047
COL4A2	ENSG00000134871	13.57121093
UBC	ENSG00000150991	11.86603618
SPTBN1	ENSG00000115306	11.86636132
COL6A2	ENSG00000142173	13.44340716
LAMC1	ENSG00000135862	11.65706539

Table SIII: Complete annotations of differentially expressed miRNAs

Many of these miRNAs belong to different clusters such as, chr9 (let-7a-1/let-7d), chr14 (miR-379/656), chr5 (miR-143/145), chr3 (miR15b/16-2), chr13 (miR17/92a-1), chr1(miR-181a-1/181b-1), chrX(miR-222/221), chr9(miR-23b/24-1), chr7(miR-29b-1/29a), chr8(miR-3150a/3150b) and may be transcribed as poly-cistronic transcripts.

Pre-miRNA	miRBASE ID	miR Family	miR Cluster
let-7a-1	MI0000060	let-7	chr9(let-7a-1/let-7d)
let-7f-1	MI0000067	let-7	chr9(let-7a-1/let-7d)
mir-1-1	MI0000651	mir-1	NA
mir-1197	MIMAT0005955	mir-370	chr14(miR-379/656)
mir-1244-2	MI0015974	mir-1244	NA
mir-1244-3	MI0015975	mir-1244	NA
mir-1276	MIMAT0005930	mir-1276	NA
mir-1302-3	MI0006364	mir-1302	NA
mir-145	MI0000461	mir-145	chr5(miR-143/145)
mir-151a	MI0000809	mir-28	NA
mir-15b	MI0000438	mir-15	chr3(miR15b/16-2)
mir-17	MI0000071	mir-17	chr13(miR17/92a-1)
mir-181a-1	MI0000289	mir-181	chr1(miR-181a-1/181b-1)
mir-222	MI0000299	mir-221	chrX(miR-222/221)
mir-223	MI0000300	mir-223	NA
mir-23b	MI0000439	mir-23	chr9(miR-23b/24-1)
mir-27b	MI0000440	mir-27	chr9(miR-23b/24-1)
mir-29a	MI0000087	mir-29	chr7(miR-29b-1/29a)
mir-3150b	MI0016426	mir-3150	chr8(miR-3150a/3150b)

mir-378a	MI0000786	mir-378	NA
mir-3945	MIMAT0018361	NA	NA
mir-3975	MIMAT0019360	NA	NA
mir-4697	MI0017330	NA	NA
mir-4768	MI0017409	NA	NA
mir-5690	MIMAT0022482	NA	NA
mir-645	MIMAT0003315	mir-645	NA
mir-873	MI0005564	mir-873	NA

Table SIV:
miRNA:mRNA and miRNA:lncRNA target correlations. lncRNA:mRNA and lncRNA:miRNA targets of miRNA as confirmed by CLIPSEQ (excel sheet)

Figure S1 a): Radar Plots of 60 women recruited in the study All the mRNA measurements for each woman are shown as individual radar plots, which are arrayed in order of the mother's ranking from the lowest (first radar) to highest (last plot). Subjects clinically in labor are shown in red while those clinically not in labor are shown in blue. Hypothesis tests of location and pairwise correlations in the non-laboring and laboring women were used for all 29 candidate genes using Wilcoxon rank-sum non-parametric tests, as appropriate to the strongly right-skewed distribution of the data using Stata 15 software (StataCorp, College Station, Texas).

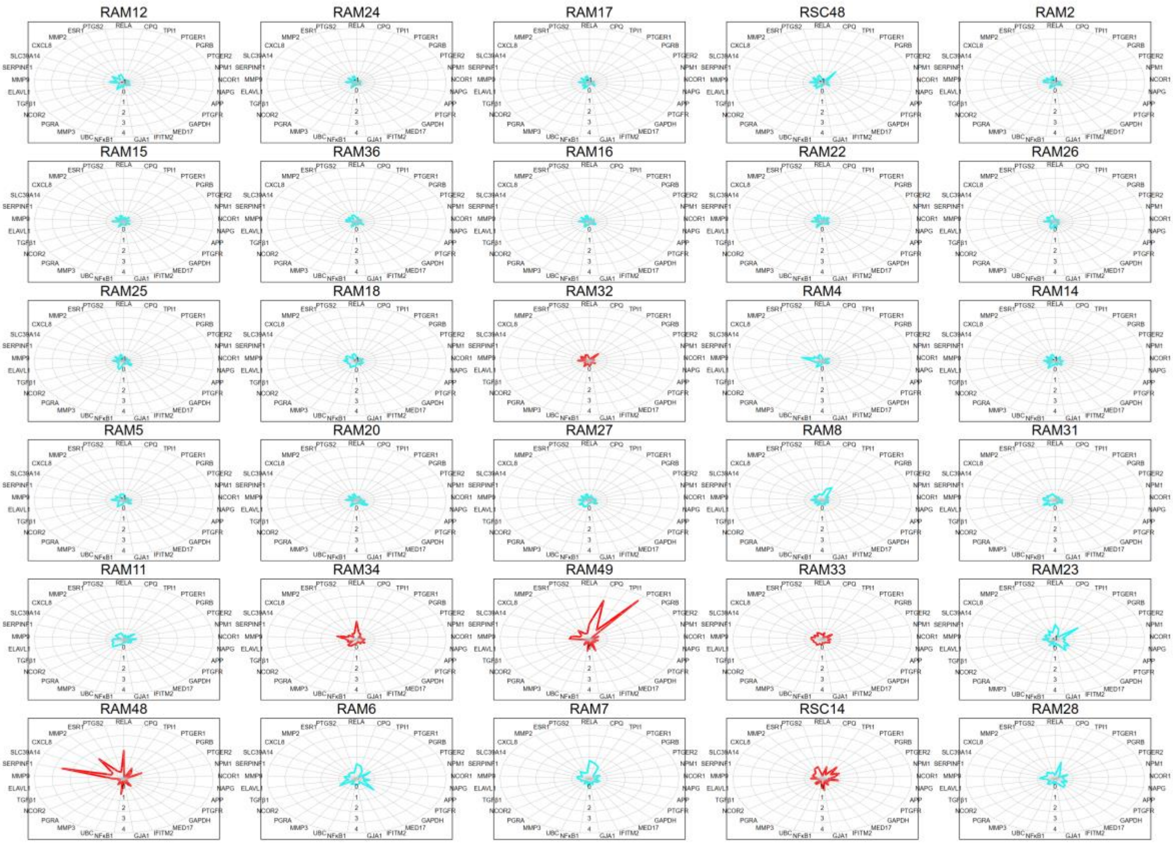
RAM12
RAM24
RAM17
RSC48
RAM2
RAM15
RAM36
RAM16
RAM22
RAM26
RAM25
RAM18
RAM32
RAM4
RAM14
RAM5
RAM20
RAM27
RAM8
RAM31
RAM11
RAM34
RAM49
RAM33
RAM23
RAM48
RAM6
RAM7
RSC14
RAM28

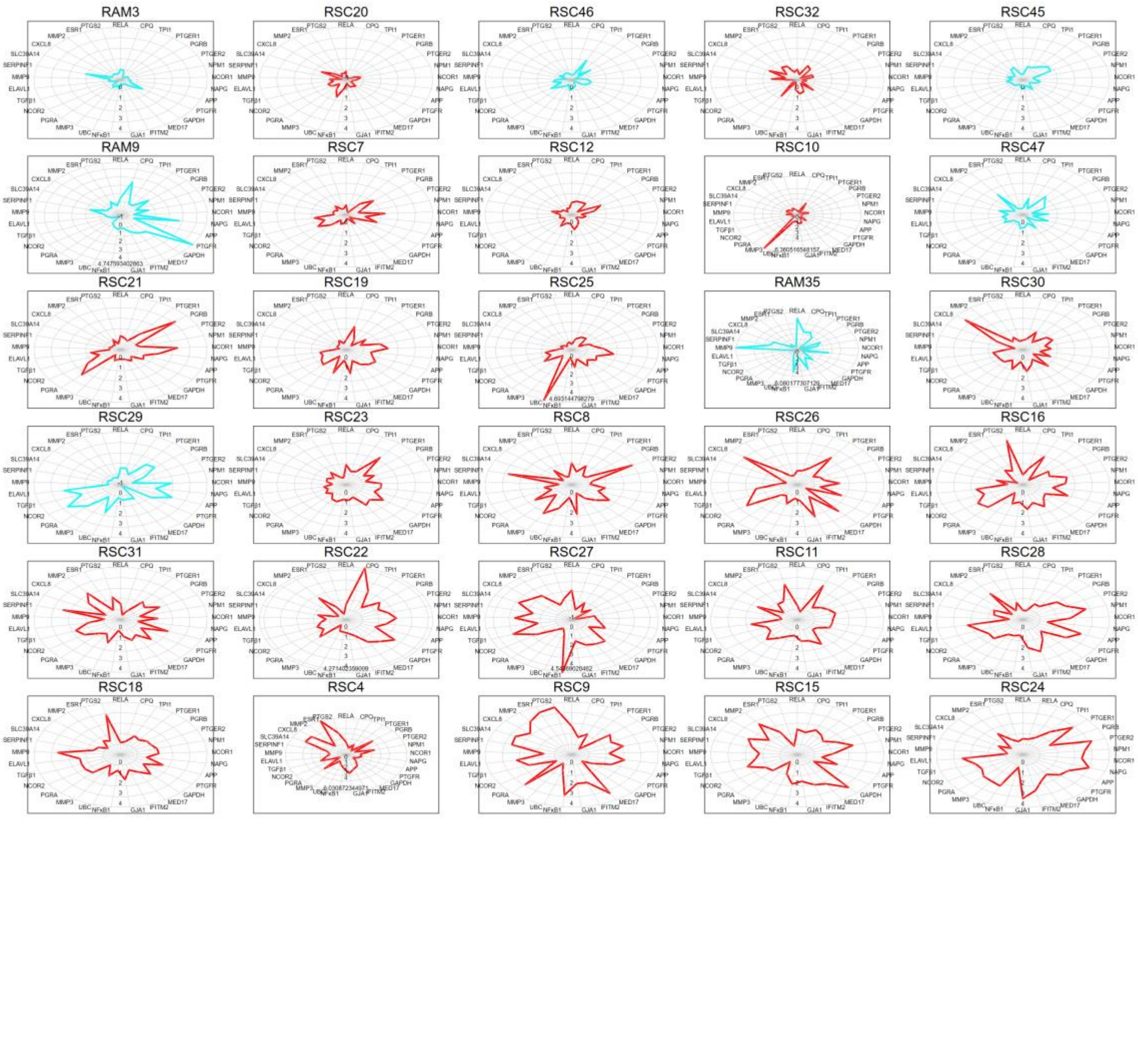
RAM3
RSC20
RSC46
RSC32
RSC45
RAM9
RSC7
RSC12
RSC10
RSC47
RSC21
RSC19
RSC25
RAM35
RSC30
RSC29
RSC23
RSC8
RSC26
RSC16
RSC31
RSC22
RSC27
RSC11
RSC28
RSC18
RSC4
RSC9
RSC15
RSC24

Figure S1 b): 3D Cigar Plots of ranked genes.

Individual radar plots were aligned in order of the maternal ranks with the lowest ranked at the front and the highest, the rear, to produce a 3-dimensional “cigar” plot. The raw data were ranked and transformed to standard normal deviates (Standardised Z-scores) for use in scatter plots and the calculation of the Pearson’s correlation coefficient. The labor status of the mothers is shown in blue for clinically non-laboring mothers and red for laboring mothers.

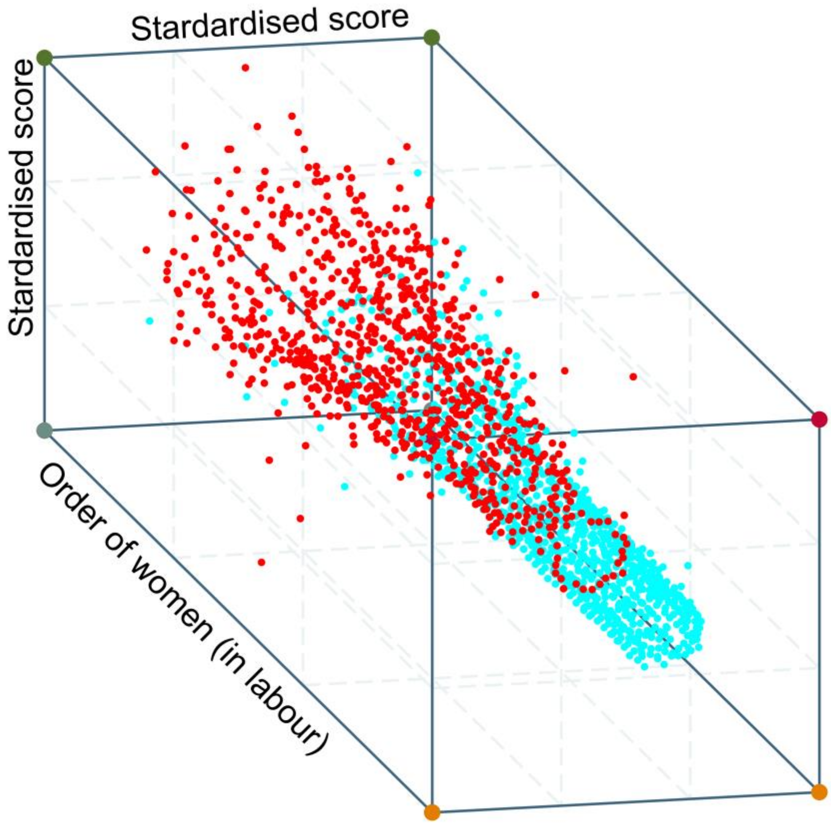

Figure S2: Hierarchical clustering of genes based on their expression profiles

a) mRNA, b) miRNA, and c) lncRNA. d) Heatmap of genes from three modules (M1, M2, M3). The three labour groups cluster together according the expression of the modules.

a

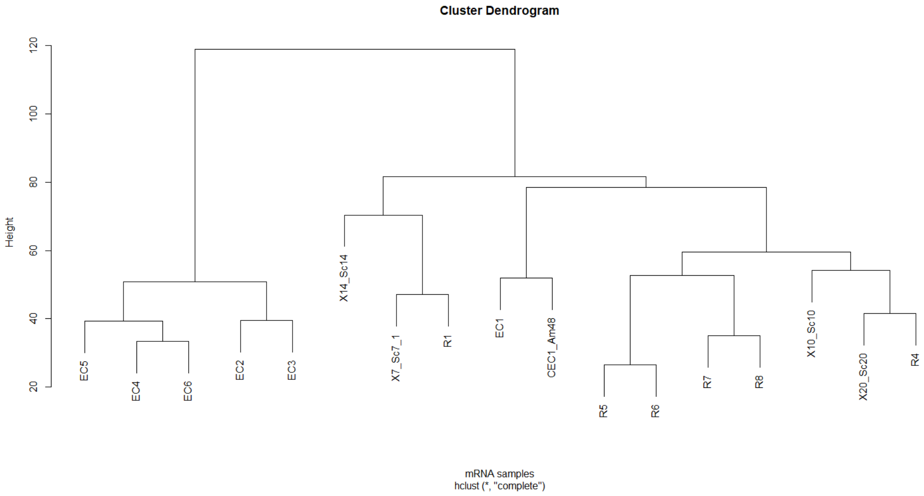

b

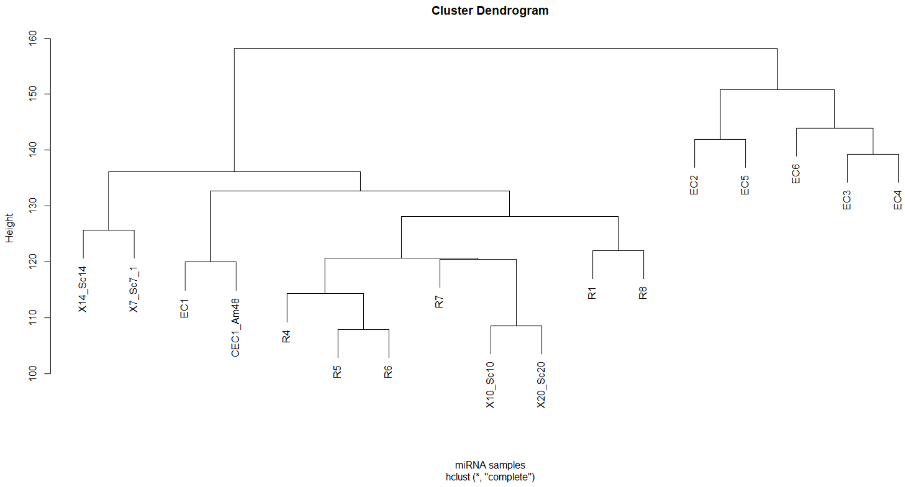

c

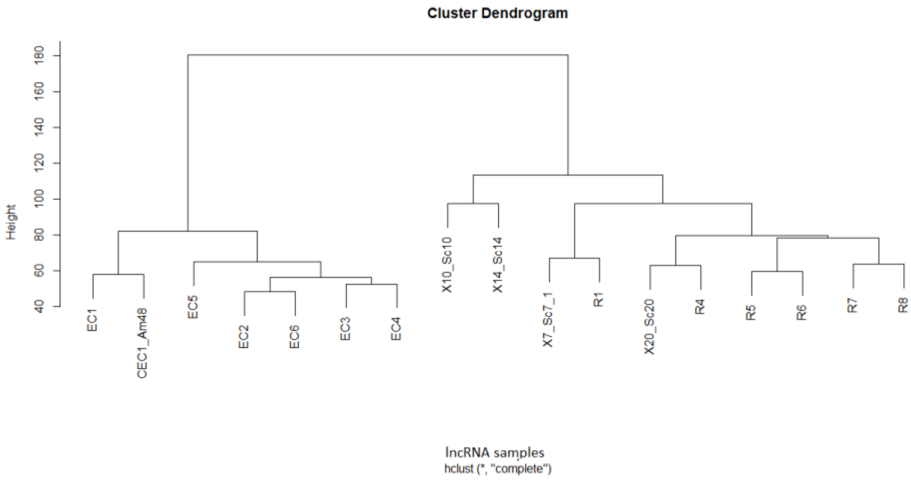

d

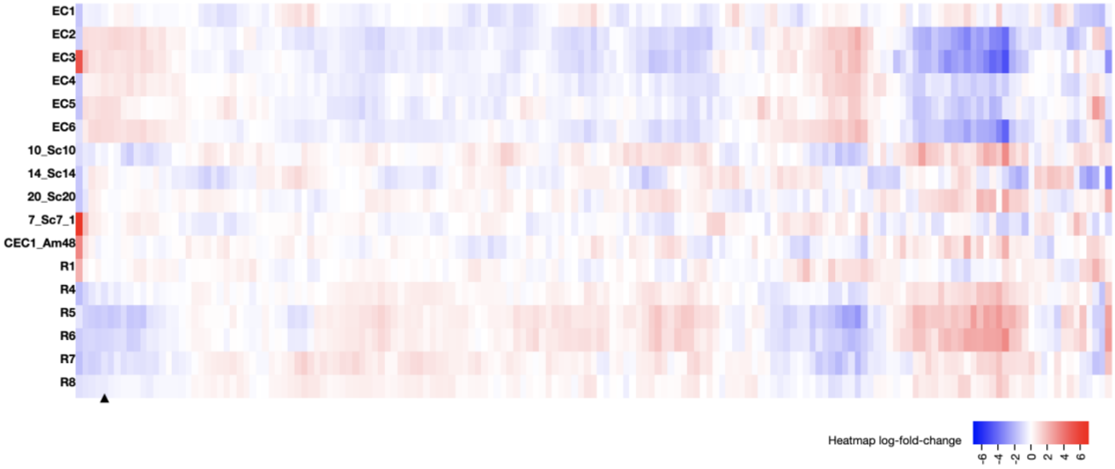

Figure S3: TFBS predicted scores trends:
Trends of Zscore, Fisher Score are not influenced by genomic GC content of the background genomes.

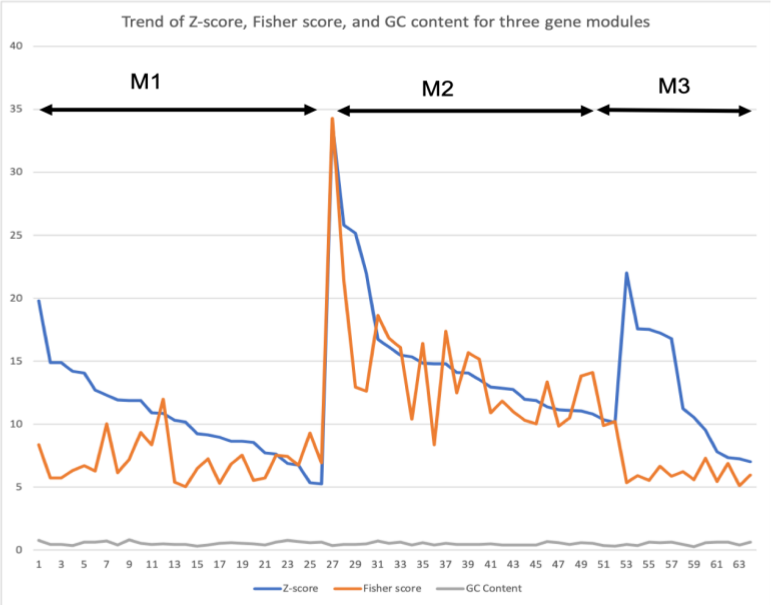

Figure S4: The KEGG pathway enrichment for all miRNA targets

The KEGG pathway enrichment for all miRNA targets as predicted by correlation analysis of mRNA and miRNA expression profiles with Pearson correlation cut off of 0.8 and adj.pavlue<0.01.

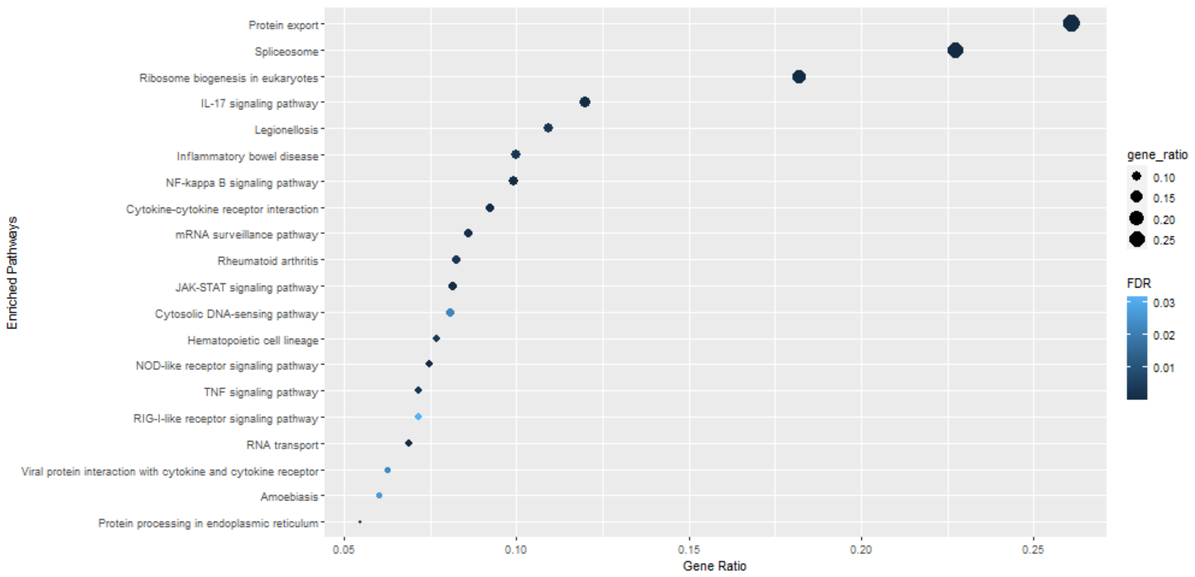

Protein export
Spliceosome
Ribosome biogenesis in eukaryotes
IL-17 signaling pathway
Legionellosis
Inflammatory bowel disease
NF-kappa B signaling pathway
Cytokine-cytokine receptor interaction
mRNA surveillance pathway
Rheumatoid arthritis
JAK-STAT signaling pathway
Cytosolic DNA-sensing pathway
Hematopoietic cell lineage
NOD-like receptor signaling pathway
TNF signaling pathway
RIG-I-like receptor signaling pathway
RNA transport
Viral protein interaction with cytokine and cytokine receptor
Amoebiasis
Protein processing in endoplasmic reticulum
Enriched Pathways
0.05
0.10
0.15
0.20
0.25
Gene Ratio
gene_ratio
0.10
0.15
0.20
0.25
FDR
0.03
0.02
0.01

Figure S5-a) Gene Set Enrichment analysis of lncRNA

Mean expression of differentially expressed lncRNA was higher than the background of the annotated lncRNAs in GENCODE v24, a) These lncRNA are located more tightly around the nearest protein coding genes than expected by chance. Lncompare database was used to perform gene set enrichment analysis.

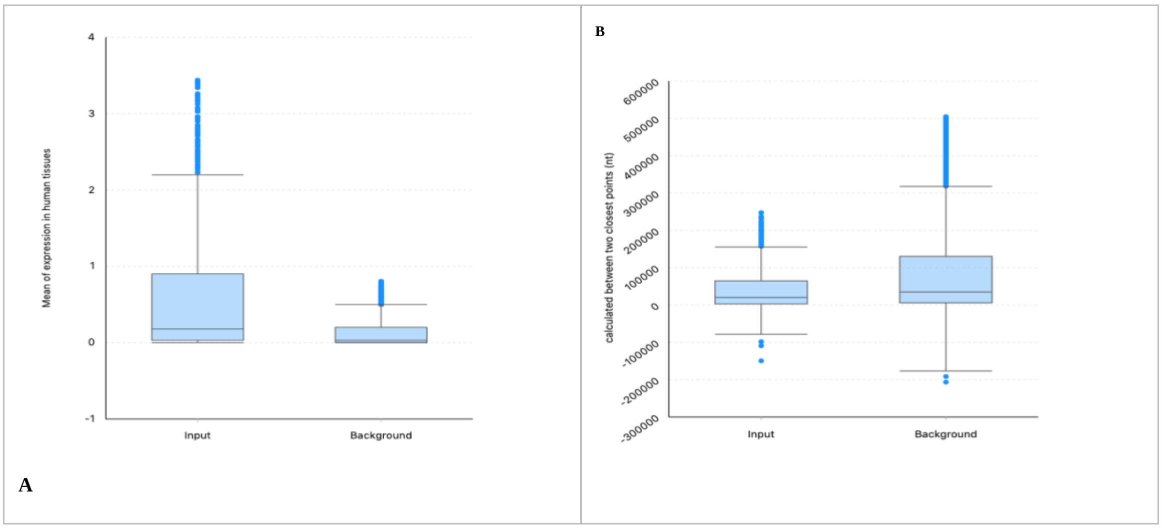

Figure S5-b): Common mRNA targets of miRNA and lncRNA

The Venn diagram represents a total of 2602 mRNA as common targets for both miRNA and lncRNA with significant negative correlation between miRNA:lncRNA (adj.pval<0.01). We are ploting mRNA that showed a positive correlation between lncRNA: mRNA, and negative correlation between miRNA:mRNA (adj.pval<0.01).

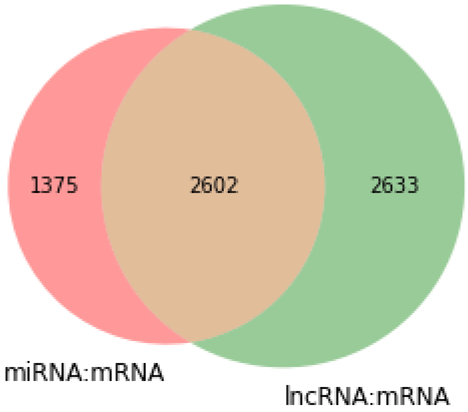

**Enriched pathways for common 2602 mRNA targets:**
rRNA modification in the nucleus and cytosol
Major pathway of rRNA processing in the nucleolus and cytosol
mRNA Splicing - Major Pathway
Cleavage of Growing Transcript in the Termination Region
Epigenetic regulation of gene expression
Cellular responses to stress
L13a-mediated translational silencing of Ceruloplasmin expression
SRP-dependent cotranslational protein targeting to membrane
